# Disseminated Hydatid Disease in a Child Involving Multiple Organ Systems: A Case Report

**DOI:** 10.7759/cureus.6564

**Published:** 2020-01-04

**Authors:** Muhammad Usman Shabbir, Awaiz Ahmed, Faizan Shaukat, Abdullah Zaki, Ghazan Askar, Iqraa Ansar, Muhammad Imran Sohail, Hamza Khan

**Affiliations:** 1 Medicine, Shifa International Hospital, Islamabad, PAK; 2 Internal Medicine, Shifa International Hospital, Islamabad, PAK; 3 Pediatrics, Shifa International Hospital, Islamabad, PAK; 4 Preventive Medicine, World Health Organization, Islamabad, PAK; 5 Surgery, Shifa College of Medicine, Islamabad, PAK

**Keywords:** parasitic infection, hydatid disease, disseminated hydatid disease, afghanistan, echinococcus, zoonosis, cystic echinococcosis

## Abstract

Hydatid disease is a parasitic infestation by Echinococcus granulosus, which involves the liver and lungs primarily. The authors report a case of disseminated hydatid disease involving multiple organs simultaneously in a 7-year-old child from Kabul, Afghanistan. The patient under examination had been having a complaint of cough and low-grade fever for the last one year. Computed tomography (CT) and ultrasonography (USG) demonstrated cystic lesions in his liver, lungs, spleen, and suprarenal region. The literature review showed that it was very rare for hydatid disease to involve multiple organs simultaneously, even in endemic areas, and the management of disseminated disease was very challenging, especially in the pediatric population.

## Introduction

Human cystic echinococcosis/hydatidosis is a dog-borne zoonosis that is caused by infection with the larval stage of the tapeworm of genus Echinococcus, namely E. granulosus and E. multilocularis. Typically, the life cycle of this parasite involves a definitive and an intermediate host. Its life cycle has three stages that encompass adult tapeworm in a definitive host; eggs in the environment and metacestodes in the intermediate host.

The life cycle begins when the metacestodes are ingested by the carnivores such as dogs (the definitive host) where they mature into the tapeworm in its intestines and are released in the form of eggs into the environment. Humans or other herbivores (the intermediate host) ingest these eggs that eventually hatch into metacestodes. They invade the blood vessels of the portal tract to migrate to the liver. Sometimes, they pass through the liver and spread to the lungs and other organs of the intermediate host [1]. In the majority of the cases, the involvement of multiple organs is a result of secondary disseminated disease. In other words, a patient has to have a prior history of hydatid disease in order to involve multiple organs. The presentation of primary dissemination through the involvement of multiple organs is very rare, especially in the pediatric population. On the whole, it is seen that exposure to food and water contaminated by the feces of an infected host or poor hygiene in areas of an infestation can cause this disease [2].

It is known that the liver is the most commonly involved organ in hydatid cyst disease [3,4]. The presence of hydatid cysts in multiple organs has not been reported as frequently. In the previous 20 years, only 463 cases of hydatid cyst in organs other than liver and lung have been reported. Nonetheless, this should always be a differential diagnosis when dealing with patients in endemic countries like India, Pakistan, Iran, and Afghanistan [1].

Disseminated presentation of the disease is rare except for the cases in which there was manipulation without caution with a sharp instrument causing the rupture of the cysts and hydatid fluid spillage, or in the pediatric group with only minimal reported cases [5].

## Case presentation

A seven-year-old boy from Kabul, Afghanistan, was evaluated by the outpatient clinic with dry cough and low-grade fever for 12 months. The fever was undocumented and associated with weakness, malaise, lethargy, loss of appetite, vague abdominal pain, and chest pain. The patient had already presented multiple times to different hospitals with similar complaints and was treated with antibiotics for an upper respiratory tract infection (URTI) with no relief.

The patient denied dry cough, bloody sputum production, and unintentional weight loss. There were no other signs of tuberculosis (TB). Moreover, no history of jaundice, bone pains, or recurrent infections were noted. Review of systems, birth, developmental, and family history was noncontributory. He was up to date with immunizations. There was a history of herding sheep and goat, which is common in Afghanistan.

On examination, the patient looked uncomfortable. Vitals: temperature 36.9 degrees Celsius (C) [98.4 Fahrenheit (F)], blood pressure 115/86, and pulse 82 per minute. The patient's weight was 50.5 pounds (lbs) [22.9 kilograms (kg)], and height was 48 inches [121.9 centimeter (cm)]. Abdominal examination revealed no increase in abdominal girth on inspection. On palpation, there was mild right upper quadrant abdominal tenderness and slight hepatomegaly (liver was detected 6 cm below costal margin). The cardiopulmonary exam was normal.

Laboratory investigation revealed Eosinophils 14% [Normal value (N): 1-2 %] and Monocytes 8% (N: 1-4%). Serology was positive for Echinococcus, indirect haemagglutinin serological test was positive. Ultrasound (USG) showed borderline hepatomegaly with cysts in both lobes, the largest one measuring 78 x 75 x 72.5 millimeter (mm) with irregular walls, a well-defined cyst of 56.7 x 52.4 mm in the left suprarenal region and small para-aortic lymph nodes ranging between 8-14 mm in size.

**Figure 1:**
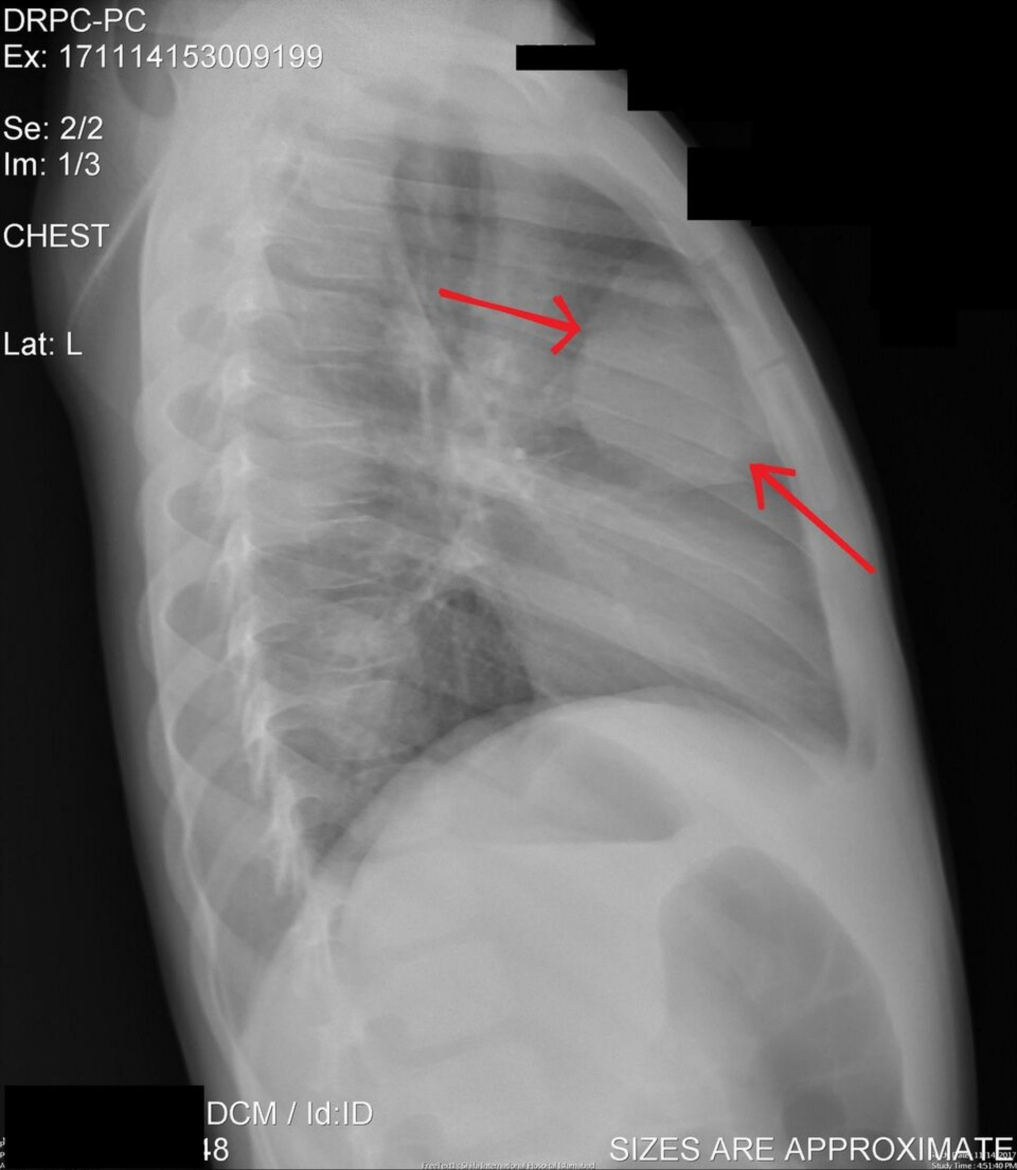
Chest X-ray (CXR) lateral view CXR lateral view showing anterior mediastinal mass.

**Figure 2:**
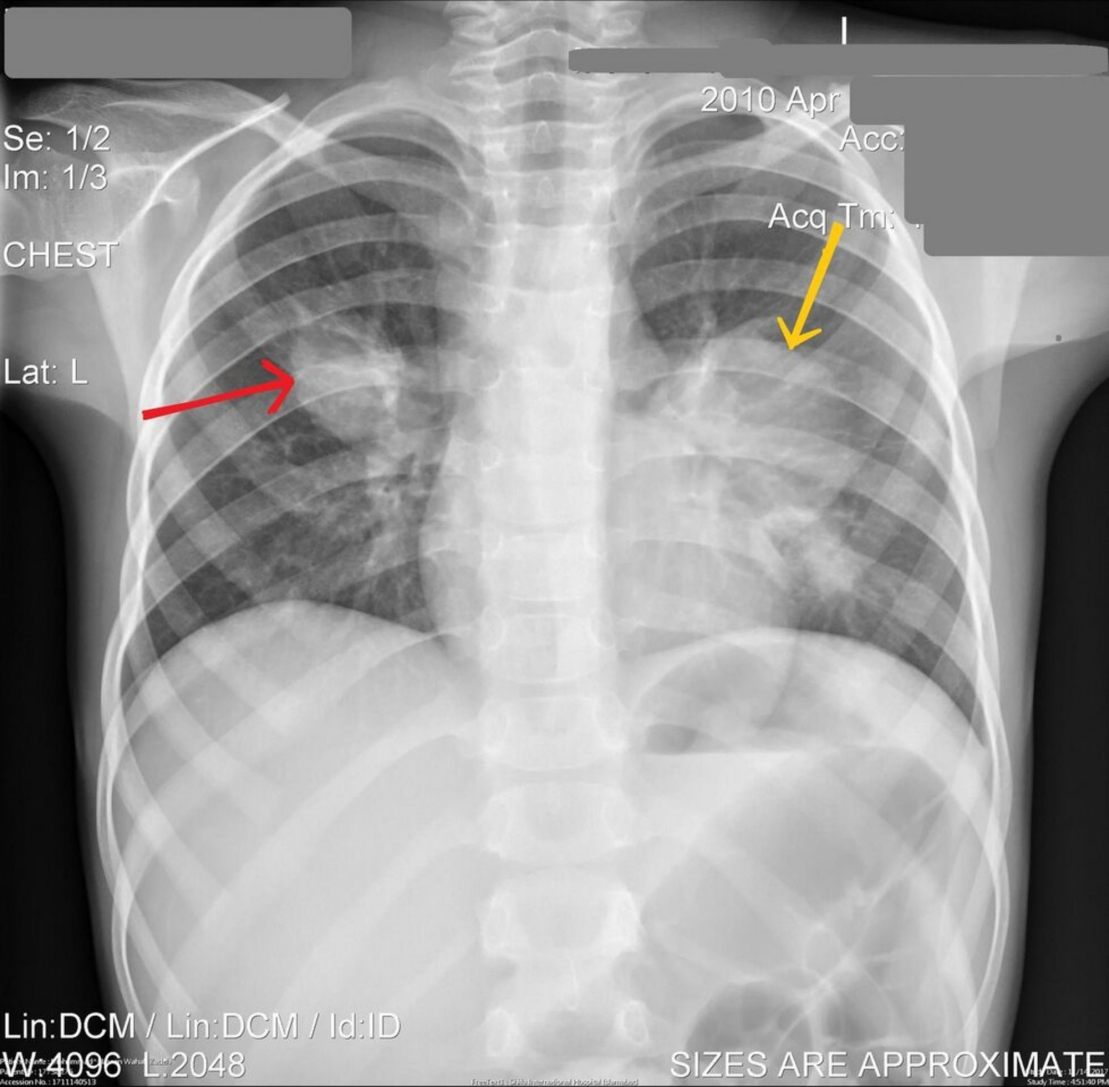
Chest X-ray anteroposterior (AP) view Arrows showing lobulated soft tissue densities in both lungs.

The chest X-ray revealed lobulated anterior mediastinal masses obliterating the retrosternal lucency, likely representing lymph node masses (Figures 1, 2).

**Figure 3:**
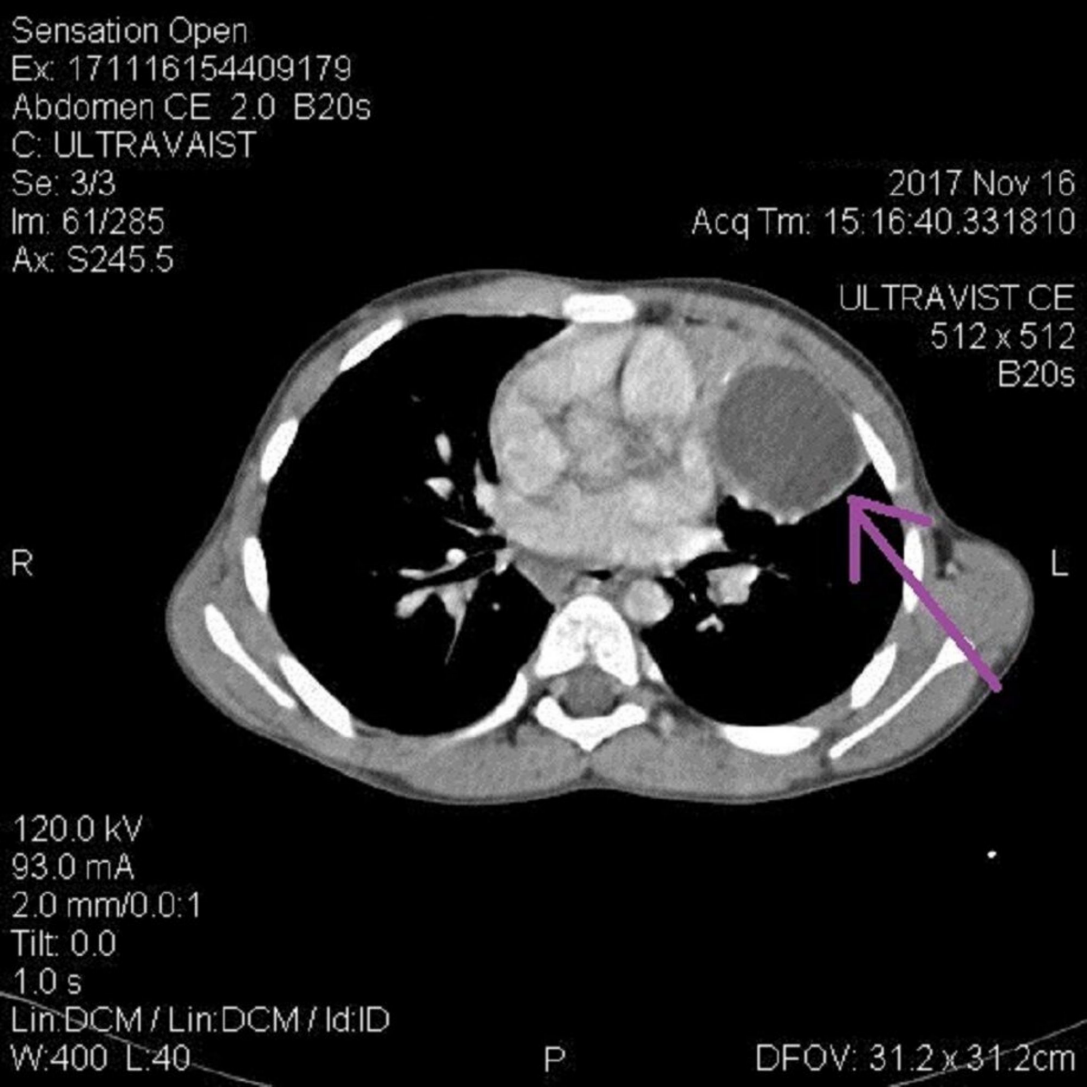
Computed tomography (CT) findings Arrow showing left lower lung lobe cyst.

**Figure 4:**
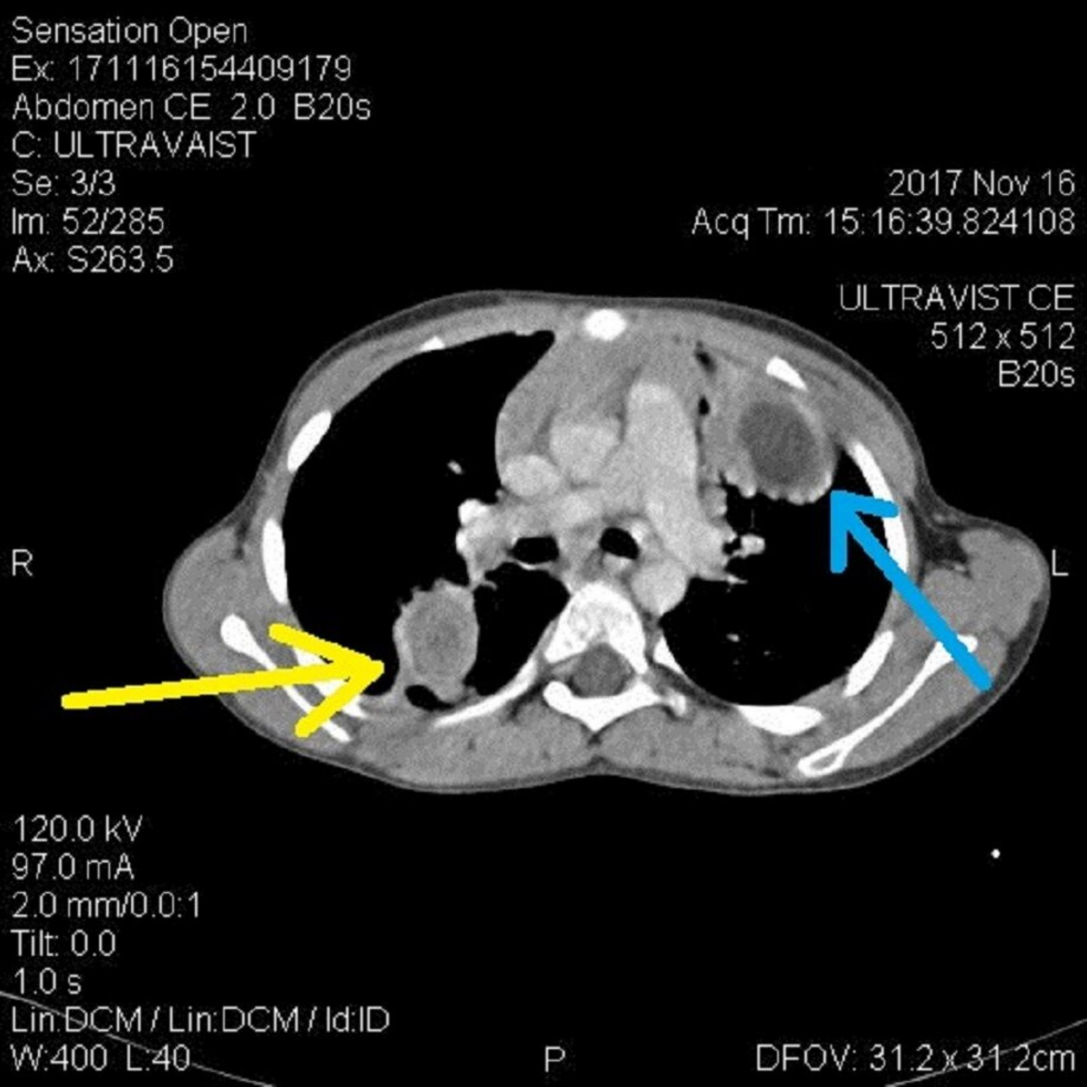
Computed tomography (CT) findings Cystic areas in left lower lung lobe (blue arrow) and apical segment of right lower lobe (yellow arrow).

Computed tomography (CT) scan showed right paratracheal lymph node measuring 10 mm along with axillary and supraclavicular lymphadenopathy. A round cystic area was noted in the left lower lung lobe measuring 50 x 44 cm (Figure 3). Another smaller round cystic area was also noted in the apical segment of the right lower lobe measuring 2.9 x 2.3 cm (Figure 4).

**Figure 5:**
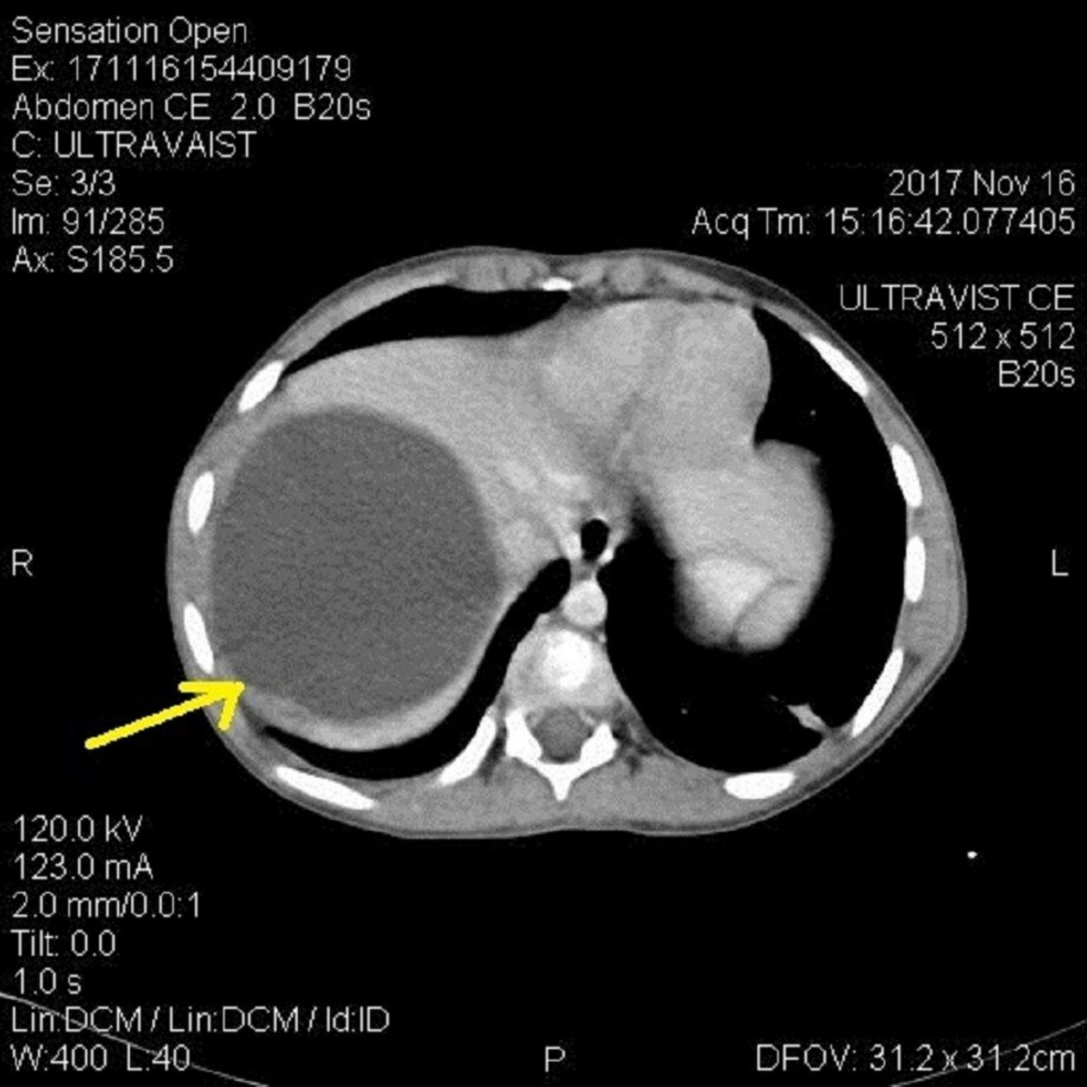
Computed tomography (CT) findings CT abdomen showing large cyst in right lobe subdiaphragmatic area of liver.

**Figure 6:**
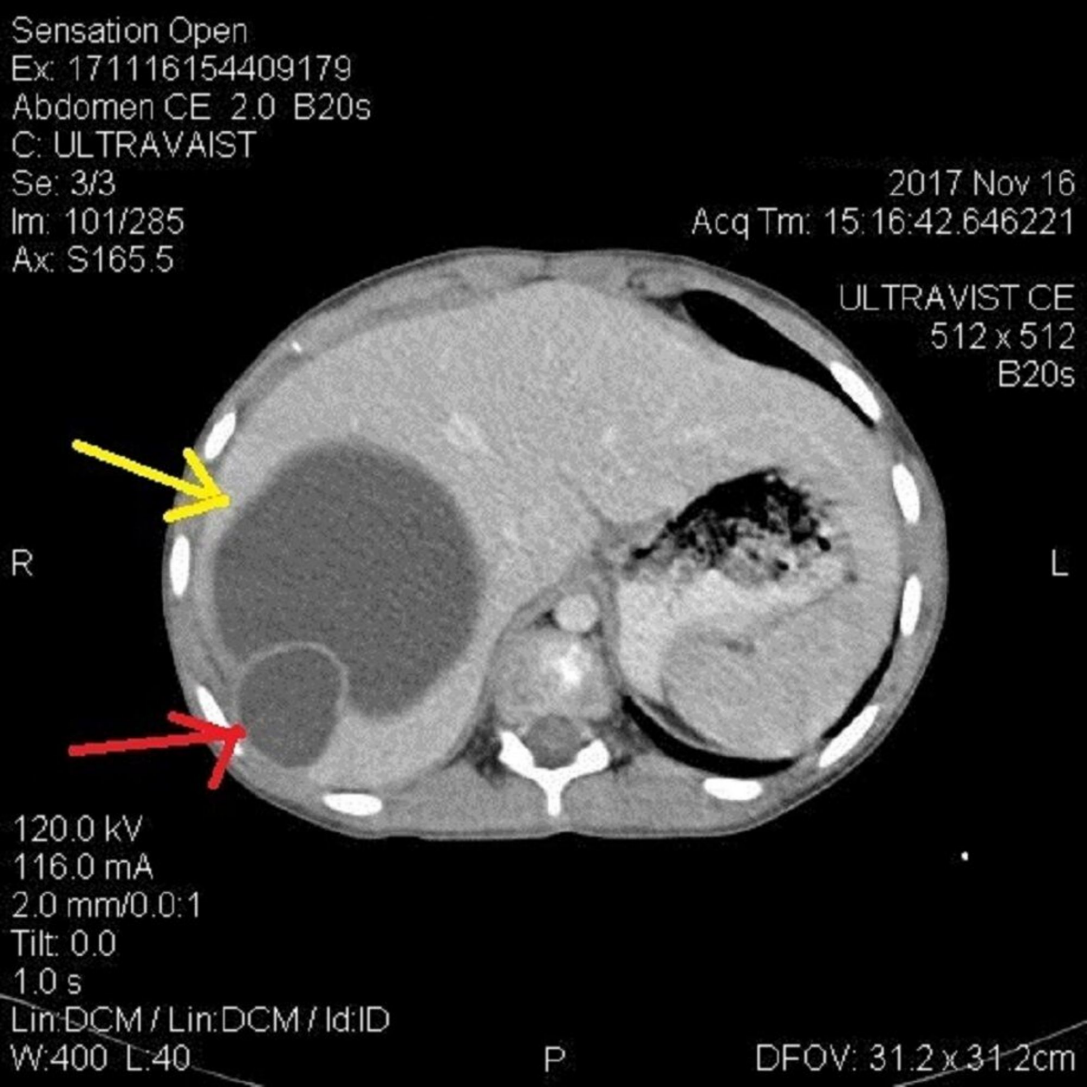
Computed tomography (CT) findings CT abdomen showing multiple cysts in liver.

**Figure 7:**
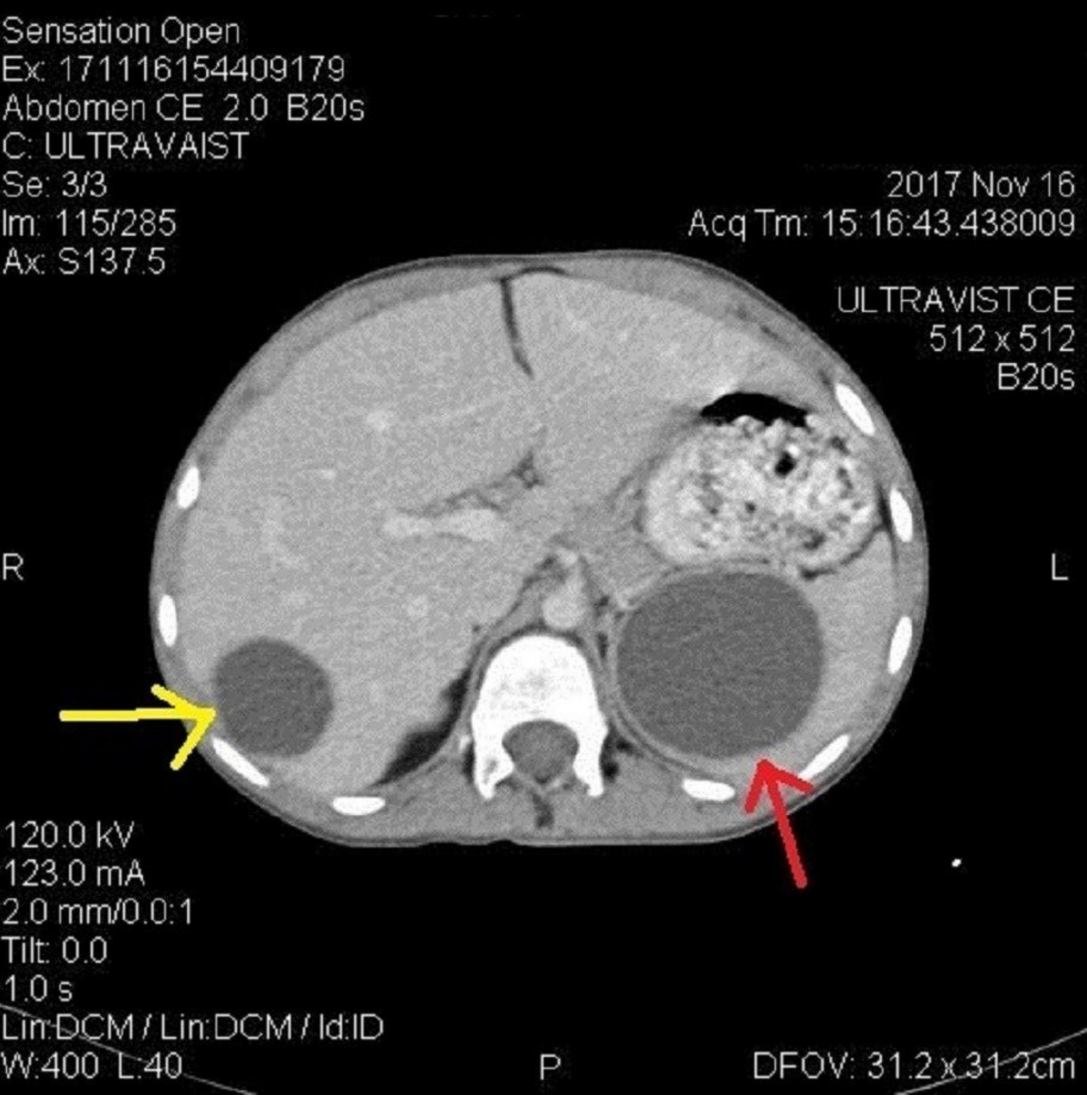
Computed tomography (CT) findings CT abdomen showing cyst in liver (yellow) and cyst in spleen (red).

The liver was mildly enlarged, and a large rounded cystic area was noted in the right lobe subdiaphragmatic area measuring 8.7 x 8.2 cm with internal fluid density (Figure 5). Similar smaller cysts were present in the right and left lobe of the liver (Figure 6). The spleen was also enlarged with a large cyst measuring 5.5 x 6 cm (Figure 7).

These findings were suggestive of hydatid cyst, and a diagnosis of disseminated hydatid disease to the lungs, liver, suprarenal gland and spleen was made. The patient was discharged on albendazole 400 milligrams (mg) twice a day, and a one-month follow-up was planned to assess for surgery. Unfortunately, the patient never returned and the case was lost to follow-up.

## Discussion

Hydatid disease is a parasitic infection by a tapeworm of the genus Echinococcus, which is caused by larval cestodes of the phylum Platyhelminthes.

Various epidemiological studies indicate that echinococcosis causes serious problems in Central Asia, which includes Mongolia, Kazakhstan, Kyrgyzstan, Tajikistan, Turkmenistan, Uzbekistan, Afghanistan, Pakistan, parts of Iran and areas of western China. More than half of the inhabitants are exposed and are at high risk of infection with E. granulosus and E. multilocularis [6]. Human hydatid cyst prevalence ranges from 1.1% to 13.7% in different parts of Iran [7]. A 1988 survey from Afghanistan showed that 73% of stray dogs in Kabul were infected with E. granulosus, given that ten times more stray dogs were present compared to house dogs in Kabul. Cases of cystic echinococcosis among Afghan immigrants have also been reported [6].

Afghanistan is a highly endemic country in regards to this disease. Even though the patient could not ascertain the correlation of definitive hosts like dogs or other carnivores, however, considering that the patient belonged to Afghanistan, the chances of hydatid cyst disease were high [6].

Gangopadhyay et al. concluded that children were more likely to have an atypical presentation of hydatid cyst disease than adults [8]. It is seen that children present with vague symptoms such as cough, lethargy, or loss of appetite. In addition, an increase in abdominal girth is also reported [3]. The pressure effects early in the course of the disease are vague. It can include symptoms of low-grade fever, nonspecific pain, cough, and sensation of abdominal fullness. However, as the mass grows, the symptoms worsen. The study by Dashti et al. looked at the clinical presentation and discovered that regardless of the site of the cyst, fever was prevalent in 68.4% of the children, and weakness was observed in 59.6% of the children [3]. In our patient, the fever and weakness prevailed for the past one year.

As for the hydatid cyst, the most common location for adults was the liver, whereas the most common location for children was the lungs. Lung involvement can be due to transdiaphragmatic migration. Our patient had cysts in his liver, lungs, spleen, and suprarenal region. The typical presentation of this disease is mainly due to mass effects or complications. The pressure symptoms are not very direct at first. For instance, a person can present with nonspecific pain, low-grade fever, cough, and the sensation of abdominal fullness [9]. In our patient, the fullness and discomfort were secondary to splenomegaly. However, hydatid splenic involvement is rare and only occurs in 1-8% of the cases [10]. Our patient also had a cough and fever since the past year with no history of any TB contact. So a cough for such a prolonged duration, which was getting worse, did signal that there was some form of pressure symptoms. Therefore, the differential diagnosis included a liver abscess, primary hepatic carcinoma, tuberculosis, and metastatic carcinoma [11]. In addition, gastrointestinal stromal tumors (GIST) could also be considered one of the differentials [12].

For diagnosis, ultrasound and CT scan are both suitable modalities. However, history, physical examination, laboratory diagnosis, and radiographic evaluation are all relevant. Considering that there are no specific clinical signs, there is a high chance of missing this diagnosis even with these modalities, as eosinophilia can be absent in some cases, and lab abnormalities might not be present. Besides, no serological or immunological test confirms the disease [9]. Even if the patient has a negative serological test, Echinococcus can still be present. In the case of our patient, the indirect haemagglutinin serological test was positive. With these findings, the test should still be compared with the radiological results, as that will give a rather clear idea about the extent of the disease and show the scale of organ involvement.

The choice of treatment varies from case to case and depends largely on the extent of the disease. Also, the size and location of the cyst present a challenge to both the physician and the surgeon. If the hydatid cyst is present in the lung and the liver, it is best treated with surgery because the patient’s symptoms are mainly due to compression. However, medical therapy is considered where patients have cysts in two or more organs and have peritoneal cysts. In the case reported by Iqbal et al. in similar settings, the patient did not undergo surgery but was given medical treatment for one year [11]. The patient responded to albendazole 400 mg (two times a day) for a year, and improvement was seen both radiologically and symptomatically. Olmez et al. also stated that medical treatment is a good choice for disseminated disease in children [13].

In the case series published by Türkyılmaz et al., medical therapy was considered in the presence of multiple cysts or disseminated disease, but unfortunately, recurrences occurred in almost all cases [14]. If a surgical approach has to be attempted, successful results were attained with cystectomy plus captionage, in which the upper edge of the cyst is sutured to the deepest part of the cavity, and a running suture collapses the other edge. This was reserved for lung cysts and partial pericystectomy with captionage with or without omentoplasty for liver cysts [14].

Although this disease can be treated both medically and surgically, some body of evidence suggests that only surgery can be a superior option. The use of short-term medical treatment after surgery has a low complication rate, and the rate of recurrence decreases as well [1].

In our case, the patient was started on medical treatment with albendazole and was called for follow-up to see how he responded. Due to the low socioeconomic status, the family wished to opt for medical treatment before any surgical intervention could be attempted. Unfortunately, this was the last correspondence with the patient and his family, and the case was lost to follow-up.

## Conclusions

The prognosis of hydatid disease depends on the initial involvement of organs. The use of medical therapy after surgery is recommended to prevent recurrence in patients but the surgery itself is a challenging task in the case of disseminated hydatid disease. Our literature review showed that it was very rare for hydatid disease to involve multiple organs simultaneously even in endemic areas and the management of disseminated disease was more challenging, especially in the pediatric population. Hydatid disease has intermediate hosts and therefore it is crucial for the transmission cycle to be broken. Nonetheless, the maintenance of hygiene and public awareness cannot be ignored.
